# Patient-specific induced pluripotent stem cell properties implicate Ca^2+^-homeostasis in clinical arrhythmia associated with combined heterozygous *RYR2* and *SCN10A* variants

**DOI:** 10.1098/rstb.2022.0175

**Published:** 2023-06-19

**Authors:** Yafei Zhou, Wenjun Huang, Leiying Liu, Anmao Li, Congshan Jiang, Rui Zhou, Jie Wang, Xiaoqiu Tan, Christopher L.-H. Huang, Yanmin Zhang

**Affiliations:** ^1^ National Regional Children’s Medical Center (Northwest); Key Laboratory of Precision Medicine to Pediatric Diseases of Shaanxi Province; Xi’an Key Laboratory of Children’s Health and Diseases, Shaanxi Institute for Pediatric Diseases, Xi’an Children’s Hospital, Affiliated Children's Hospital of Xi'an Jiaotong University, No. 69, Xijuyuan Lane, Xi'an 710003, People's Republic of China; ^2^ Department of Cardiology, Xi'an Children's Hospital, Affiliated Children's Hospital of Xi'an Jiaotong University, No. 69, Xijuyuan Lane, Xi'an 710003, People's Republic of China; ^3^ Key Laboratory of Medical Electrophysiology of the Ministry of Education, and Medical Electrophysiological Key Laboratory of Sichuan Province, Institute of Cardiovascular Research, Southwest Medical University, Longmatan District, Luzhou 646000, People's Republic of China; ^4^ Physiological Laboratory, Department of Biochemistry, University of Cambridge, Cambridge CB2 3EG, UK

**Keywords:** induced pluripotent stem cell, polymorphic ventricular tachycardia, *RYR2*, *SCN10A*

## Abstract

We illustrate use of induced pluripotent stem cells (iPSCs) as platforms for investigating cardiomyocyte phenotypes in a human family pedigree exemplified by novel heterozygous RYR2-A1855D and SCN10A-Q1362H variants occurring alone and in combination. The proband, a four-month-old boy, presented with polymorphic ventricular tachycardia. Genetic tests revealed double novel heterozygous RYR2-A1855D and SCN10A-Q1362H variants inherited from his father (F) and mother (M), respectively. His father showed ventricular premature beats; his mother was asymptomatic. Molecular biological characterizations demonstrated greater *TNNT2* messenger RNA (mRNA) expression in the iPSCs-induced cardiomyocytes (iPS-CMs) than in the iPSCs. Cardiac troponin Ts became progressively organized but cytoplasmic RYR2 and SCN10A aggregations occurred in the iPS-CMs. Proband-specific iPS-CMs showed decreased *RYR2* and *SCN10A* mRNA expression. The RYR2-A1855D variant resulted in premature spontaneous sarcoplasmic reticular Ca^2+^ transients, Ca^2+^ oscillations and increased action potential durations. SCN10A-Q1362H did not confer any specific phenotype. However, the combined heterozygous RYR2-A1855D and SCN10A-Q1362H variants in the proband iPS-CMs resulted in accentuated Ca^2+^ homeostasis disorders, action potential prolongation and susceptibility to early afterdepolarizations at high stimulus frequencies. These findings attribute the clinical phenotype in the proband to effects of the heterozygous *RYR2* variant exacerbated by heterozygous *SCN10A* modification.

This article is part of the theme issue ‘The heartbeat: its molecular basis and physiological mechanisms’.

## 1. Introduction

Inherited pro-arrhythmic cardiac channelopathies have been associated with, among others, gene variants altering intracellular sarcoplasmic reticular (SR) Ca^2+^ store release and action potential (AP) properties and waveform. These have involved channel proteins including those encoded by *RYR2*, *CALM1*, *CALM2* and *KCNJ2* and *SCN5A* [1,2]. Among these, the ryanodine receptor (RYR2) acts as an intracellular SR Ca^2+^ release channel key to cardiac excitation–contraction coupling [3]. Dominant gain of function *RYR2* variants occur in 60–70% of patients with the autosomal dominant pro-arrhythmic condition catecholaminergic polymorphic ventricular tachycardia (CPVT) [4]. Patients with *RYR2* variants also present with multiple phenotypes including sustained ventricular tachycardia, idiopathic ventricular fibrillation, atrial fibrillation (AF) and cardiomyopathies [5,6]. The underlying *RYR2* variants may either increase channel sensitivity to SR luminal Ca^2+^ causing channel opening at reduced intra-SR [Ca^2+^] or result in a tendency to store overload-induced Ca^2+^ release. The enhanced SR diastolic Ca^2+^ release associated with *RYR2* gain of function causes an intracellular Ca^2+^ overload during exercise or emotional stress [7]. Extrusion of the excess Ca^2+^ by the bidirectional (3Na^+^ : 1Ca^2+^) electrogenic Na^+^/Ca^2+^ exchanger (NCX) [8] predisposes to early afterdepolarizations (EAD) or delayed afterdepolarization (DAD) events potentially triggering arrhythmia [9]. Genetic analysis demonstrates that CPVT patients may have more than two gene variants; Roston *et al*. [10] demonstrated that about 8% of 237 CPVT patients showed *RYR2* variants plus variants in other CPVT-linked genes. In addition, *SCN5A*, *TRDN* and *CALM1-3* have also been implicated in CPVT and these mutations can affect cardiomyocyte APs and calcium transients [11,12].

*SCN5A* encodes the major, canonical, cardiac voltage-gated sodium channel Na_V_1.5 causing the AP up-stroke. However, abnormalities in several independent *SCN10A* loci are also clinically associated with altered cardiac conduction, AF [13,14] and Brugada syndrome (BrS) [15,16]. Nav1.8 is the neuronal sodium channel type, which has been found in some neurons such as dorsal root, cranial sensory ganglia, as well as in cardiac nerves and ganglionated plexi [17,18]. The role of Na_V_1.8 channels in late sodium current (*I*_Na-L_) in cardiomyocytes has been suggested in functional studies. *SCN10A* variants also had been associated with epileptic encephalopathy, congenital pain syndromes and neuromuscular disease [19,20]. There is a high degree of linkage disequilibrium between the common genetic variants in *SCN10A* and these mutations modulate the activity of an enhancer that controls neighbouring *SCN5A* gene expression [21]. In *SCN10A*, common genetic variants influence the PR interval, P-wave, and QRS complex. These imply potential pro-arrhythmic impacts of *SCN10A* [22].

This study describes use of induced pluripotent stem cells (iPSCs) to determine cardiomyocyte phenotypes in a family pedigree including novel heterozygous RYR2-A1855D and SCN10A-Q1362H variants occurring alone and in combination. The proband was a four-month-old boy diagnosed with CPVT. Genetic tests revealed two novel heterozygous RYR2-A1855D and SCN10A-Q1362H variants inherited from his father and mother respectively. We investigated the hypothesis that the double variants resulted in an enhanced arrhythmogenic effect. We investigated its possible basis in altered electrophysiology and Ca^2+^ homeostasis in cardiomyocytes derived from iPSCs generated from the proband and his parents. The findings bear on the possible functions of Nav1.8 and its functional relationships with RYR2.

## 2. Material and methods

### (a) Family pedigree

**Figure 1.**
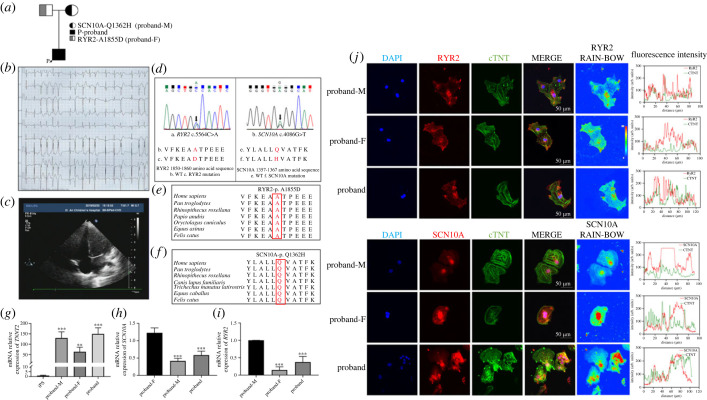
Proband information from the family pedigree showing PVT and characterization of the iPSCs-induced cardiomyocytes (iPS-CMs). (*a*) The family pedigree. (*b*) The ECG of the proband. (*c*) The ultrasonic cardiogram (UCG) of the proband. (*d–f*) Variant information on the family pedigrees (RYR2-A1855D and SCN10A-Q1362H). (*g*) The relative messenger RNA (mRNA) expression of *TNNT2* in iPS-CMs of proband-M, proband-F and proband compared with proband-M iPSCs. (*h*) The relative mRNA expression of *SCN10A* in iPS-CMs of proband-M and proband compared with proband-F. (*i*) The relative mRNA expression of *RYR2* in iPS-CMs of proband-F and proband compared with proband-M. (*g*–*i*) *p < 0.05, **p < 0.01, ***p < 0.001. Data shown are mean fold change ± s.e.m., *n* = 4, *p < 0.05, one-way ANOVA. (*j*) The location of RYR2, SCN10A and cardiac troponin T (cTNT) arrangement in iPS-CMs of the proband-M, proband-F and proband. Scale bars, 50 µm.

The investigation was conducted following principles defined by the Helsinki Declaration and approved by the Ethics Committee of Xi'an Children's Hospital (no. 2019-599), Affiliated Children's Hospital of Xi'an Jiaotong University. The family pedigree (figure 1*a*) of a four-month-old boy with CPVT was obtained from the Department of Cardiology, Xi'an Children's Hospital in 2019. Clinical phenotypes of the pedigree were deduced from the clinical history and physical, electrocardiographic (ECG) and ultrasoundcardiographic (UCG) examination. Written informed consents were obtained from the parents. We term the proband father as proband-F and the proband mother as proband-M.

### (b) Genetic analysis

Genomic DNA was extracted and clinical whole exons were tested using next generation sequencing target capture (SinoPath Company, China). The pathological characteristics of suspicious variants were predicted using multiple bioinformatics software, including Polyphen-2, Provean, SIFT and Variant Taster. The clinical significance of detected variants was assessed following the American Society of Medical Genetics and Genomics guidelines.

### (c) Generation of induced pluripotent stem cells and induced pluripotent stem cells-induced cardiomyocytes differentiation

iPSCs were generated from primary human peripheral blood mononuclear cells. The cells were generated in feeder-free culture conditions using the integration-free CytoTune-iPS 2.0 Sendai Reprogramming Kit (cat. no. A16517, Thermo Fisher Scientific, USA) with the reprogramming factors OCT4, KLF4, SOX2, c-MYC, according to manufacturer's instructions with modifications [23] (electronic supplementary material, figure S1). iPSCs-induced cardiomyocytes (iPS-CMs) were induced by the widely used GiWi-protocol originally proposed by Liu *et al*. [24]. The iPS-CMs usually beat from 10 to 12 days after differentiation.

### (d) Quantitative polymerase chain reaction

Total RNA was extracted from cells using TRIzol Reagent (Invitrogen, Waltham, MA, USA). cDNA was synthesized using the 5 × PrimeScript RT Master Mix (cat. no. RR036A-1, Takara, Japan) as follows: 37°C for 15 min; 50°C for 5 min and 98°C for 5 min. Real time-quantitative polymerase chain reaction (RT-qPCR) was performed on the platform of the CFX Connect Real-Time System (BIO-RAD, USA) using 2 × TB Green Faster qPCR Mix (Cat. no. RR430, TaKaRa, Japan) as follows: 95°C for 10 s; 52°C for 10 s and 72°C for 10 s for 40 cycles. All primers are listed in the electronic supplementary material, table S2. The expression levels of messenger RNA (mRNA) were normalized to those of *GAPDH* and calculated using the 2^−ΔΔCt^ method.

### (e) Immunofluorescence staining

Immunofluorescence staining was performed using appropriate primary antibodies and Alexa Fluor conjugated secondary antibodies. Cells were washed with pre-cooled Dulbecco's phosphate buffered saline three times and fixed with 4% paraformaldehyde at room temperature (RT) for 10 min. Then cells were permeabilized with 0.5% Triton X-100 (cat. no. A600198-0500, BBI, China) for 20 min. Cells were blocked with 2.5% bovine serum albumin (BSA; Sigma Aldrich, Cat. no. F7524) in phosphate buffered saline (PBS) at RT for 1.5 h. Primary antibodies to RYR2 (cat. no. 19766-1-AP, Proteintech, China) and SCN10A (cat. no. 33722A18, Invitrogen, USA) were applied in 1% BSA overnight at 4°C.

After being washed with DPBS for 10 min, cells were stained with diluted secondary antibody Alexa FluorTM 488 F(ab’)2 fragment of goat anti-Rabbit lgG (H + L) (cat. no. A11070, Invitrogen, USA) and Alexa FluorTM 555 F(ab’)2 fragment of goat anti-mouse lgG (H + L) (cat. no. A21425, Invitrogen, USA) (1 : 500 in DPBS), incubated for 1 h avoiding light at RT followed by counterstaining with 4', 6-diamidino-2-phenylindole DAPI (cat. no. D1306, ThermoFisher, USA) for 10 min. Fluorescence images were captured with the Olympus Fluoview system (FV3000, Olympus, Japan) to assess *in vitro* expression.

### (f) Measurements of cellular Ca^2+^ homeostasis

iPS-CMs were seeded on Matrigel-coated 14 mm glass bottomed dishes for 3–5 days and Ca^2+^ transients were measured when cells resumed beating. iPS-CMs were loaded with the cell permeable fluorescent Ca^2+^ indicator in media containing 2 µM of Fluo-4 AM (cat. no. F14201, Thermo Fisher Scientific) and 0.02% F-127 (cat. no. P3000MP, Thermo Fisher Scientific) for 10 min. During imaging, the dishes were kept in a heated 37°C stage bottomed environmental chamber and Ca^2+^ transients from single beating iPS-CMs were measured using Zeiss LSM 980 in line-scan mode. After finishing baseline recordings, appropriate amounts of 100 nM isoproterenol (ISO) (cat. no. I2760, Merck, Germany) were added into the recording dish dropwise. Fluorescent signals were normalized to the baseline cellular fluorescence (*F*_0_).

### (g) Electrophysiological studies

Briefly, at 40 days after iPS-CM differentiation, the iPS-CMs were dissociated into single cell suspension and transferred to a temperature-controlled chamber (MappingLab, USA). APs were measured at 37°C using a modified Tyrode's solution containing (in mM) 140 NaCl, 5.4 KCl, 1.8 CaCl_2_, 1.0 MgCl_2_, 5.5 glucose, 5 HEPES, pH 7.4 (NaOH). Pipettes were filled with (in mM) 125 K-gluconate, 20 KCl, 5 NaCl, 0.44 amphotericin-B, 10 HEPES, pH 7.2 (adjusted using potassium hydroxide (KOH)), and osmolality 290 ± 3 mOsm. Electrodes were fabricated from borosilicate glass (World Precision Instruments, Sarasota, FL, USA) with tip resistances of 4.5–6.5 MΩ when filled with internal solution containing (in mM): 110 K-gluconate, 20 KCl, 1 CaCl_2_, 1 MgCl_2_, 10 HEPES, 5 EGTA-KOH, 5 ATP-Mg^2+^, and 5 Na-phosphocreatine. The pH was adjusted to 7.2 by KOH, and the osmolality to 290 ± 3 mOsm. An Axon 700B amplifer (Axon Instruments, USA) was used for recordings, and the signals were digitized by a Digidata 1550B A/D converter (Axon Instruments, USA) under software control (pClamp10.4, USA).

### (h) Statistical analysis

Data are presented as mean ± s.e.m. Statistical significance of differences for normally distributed data was tested by unpaired Student's *t*-test to compare two groups and ANOVA with Tukey's *post hoc* test to compare multiple groups. Data were analysed by GraphPad Prism 8 and differences were deemed as statistically significant if *p* < 0.05.

## 3. Results

### (a) Clinical characteristics of the family pedigree

The proband was admitted to hospital presenting with increased heart rate following diarrhoea. His ECG showed multiple episodes of polymorphic ventricular tachycardia (PVT) (figure 1*b*). The administration of lidocaine, amiodarone and multiple electrical cardioversions all proved ineffective. After 2 days with the further addition of oral digoxin therapy, ECG monitoring demonstrated frequent premature ventricular beats and shortened paroxysmal ventricular tachycardic (VT) episodes. Following maintenance using propafenone and metoprolol, sinus rhythm was gradually restored (figure 1*c*). Figure 1*d*–*f* summarizes background information regarding the proband RYR2-A1855D and SCN10A-Q1362H combined heterozygous variants (figure 1*d*), and confirms the conservative nature of the RYR2-A1855 (figure 1*e*) and SCN10A-Q1362 (figure 1*f*) residues.

Admission laboratory tests showed elevated proband hypersensitive troponin (cardiac troponin T (cTNT)) (30.82 pg ml^−1^, normal range less than 0.03 ng ml^−1^) and brain natriuretic peptide precursor levels (20802.00 pg ml^−1^, normal range less than 300 pg ml^−1^). These fell to 15.06 pg ml^−1^ and 2414.00 pg ml^−1^, respectively, after VT remission. UCG revealed patent foramen ovale, horizontal left to right atrial shunting and little to moderate bicuspid and tricuspid valve regurgitation which returned to normal during follow-up. Propranolol was applied and there has been no reported syncope and ventricular tachycardic incidents in the proband in the 3 years follow-up.

Whole exon tests demonstrated that the proband carried the novel heterozygous variants of RYR2-A1855D, in which alanine (A) at position 1855 was mutated to aspartic aid (D) and SCN10A-Q1362H in which glutamine (Q) at position 1362 was mutated to histidine (H), missense variants inherited from the father and mother, respectively (figure 1*d*). A1855 is a nonpolar amino acid with hydrophobic side chains; the alanine variants may affect the protein N-terminal domain [25]. Q1362 is a polar amino acid with hydrophilic side chains [26]; a conserved glutamine may function in voltage-gated Ca^2+^ channels [27]. However, analysis of structural change alone may not be sufficient to analyse the protein functional alterations (figure 1*e*,*f*). Proband-F had a history of occasional ventricular premature beats without syncope. Proband-F's corrected QT interval (QTc) maximum was 440 ms. Proband-M was and remains asymptomatic. Both proband-F and proband-M showed normal cardiac structure.

### (b) Characterization of induced pluripotent stem cells, and induced pluripotent stem cells-induced cardiomyocytes

Detailed characteristics of the proband iPSCs have been reported on previous occasions [28]. Authenticity of the parents' iPSCs lines is illustrated in the electronic supplementary material, figures S1 and S2. The iPSCs showed strongly positive OCT4 expression (electronic supplementary material, figures S1 and S2A,B), as well as the trilineage differentiation potential. Sanger sequencing confirmed the variant of *RYR2* and *SCN10A* in the mutant clones from the family pedigree iPSCs lines (electronic supplementary material, figures S1 and S2C). The iPSCs markers were further confirmed using flow cytometry analysis (electronic supplementary material, figure S1D): the TRA1-60, SSEA4 and OCT4 positive proportions of proband-M were 87.64%, 97.11% and 94.21%, respectively. The karyotype analysis of the proband-M is shown in the electronic supplementary material, figure S1E. The TRA1-60, SSEA4 and OCT4 positive proportions of proband-F were 98.33%, 98.63% and 99.58%, respectively (electronic supplementary material, figure S2D). The karyotype analysis of proband-F is shown in the electronic supplementary material, figure S2E. The short tandem repeat detection of the parents is shown in the electronic supplementary material, figures S3 and S4, and table S1. Both mycoplasma and sendai viral vectors detection of iPSCs were detected negative (electronic supplementary material, figures S5 and S6).

Figure 1*g–j* confirms specific features of the iPS-CMs. Cardiomyocyte-specific troponin T2 (*TNNT2*) expression was significantly increased in iPS-CMs compared with that in the iPSCs (figure 1*g*). *SCN10A* expression in iPS-CMs of the proband-M (0.42 ± 0.04) and the proband (0.56 ± 0.06) was significantly lower than that of the proband-F (1.23 ± 0.07, *p* < 0.001) (figure 1*h*). The *RYR2* mRNA expression of proband and the proband-F were significantly decreased (to 0.38 ± 0.08 and 0.15 ± 0.05; respectively) (figure 1*i*). Immunofluorescence demonstrated a positive striated pattern distribution of the cardiomyocyte marker cTNT (figure 1*j*). Quantitative immunofluorescence analysis demonstrated that the RYR2-A1855D and SCN10A-1362H variants resulted in protein aggregation in the cytoplasm (figure 1*j*).

### (c) The combined *RYR2* and *SCN10A* heterozygous variants alter cellular Ca^2+^ homeostasis

Cytosolic Ca^2+^ concentration [Ca^2+^]_i_ is a ubiquitous intracellular second messenger. Pathological unregulated SR Ca^2+^ release is a potential cause of life-threatening arrhythmias [29]. Occurrence of premature spontaneous diastolic SR Ca^2+^ transients (PCT) activating Na^+^/Ca^2+^ exchange, and generation of DAD events and repetitive increases and decreases in [Ca^2+^]_i_ termed Ca^2+^ oscillations (COs) [30] are considered to indicate high tachycardic risks for CPVT [31].

**Figure 2.**
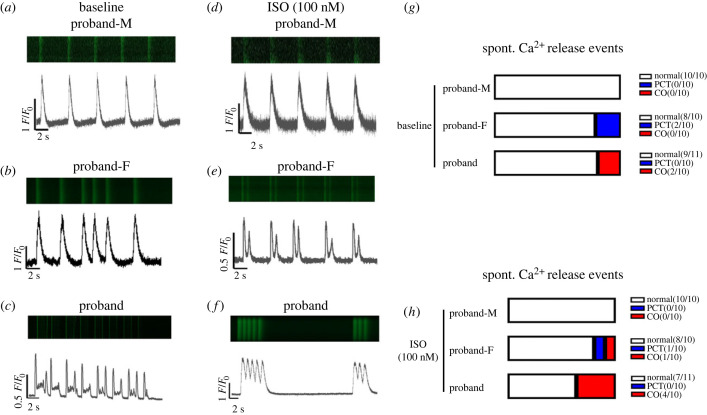
Ca^2+^ transient recordings from iPS-CMs. (*a–c*) Representative Ca^2+^ signal traces of spontaneously beating iPS-CMs in baseline spatially averaged over confocal line scans within individual iPS-CMs in cell clusters. (*d–f*) Representative Ca^2+^ signal traces of spontaneously beating iPS-CMs under isoproterenol (ISO) challenge. (*g*,*h*) Quantitative analysis of results. Numbers by blue colours denote number of cells examined that showed premature spontaneous SR Ca^2+^ transients. Numbers by red colours denote number of cells examined that showed Ca^2+^ oscillations.

**Figure 3.**
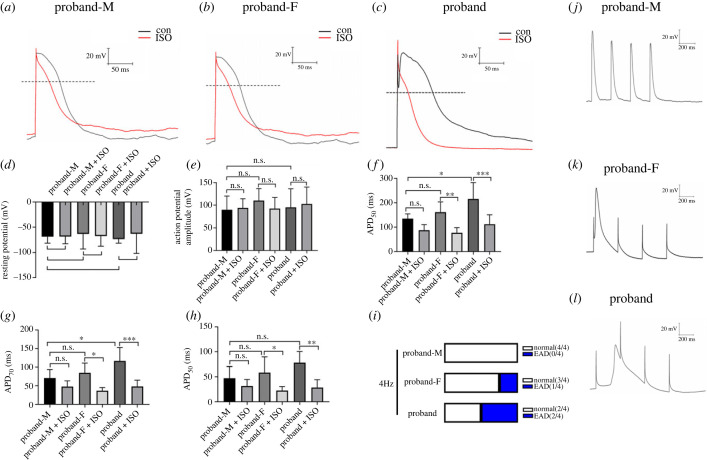
The action potential properties of iPS-CMs. (*a–c*) AP waveforms recorded using whole cell patch electrodes at 1 Hz stimulation for proband-M (*a*), proband-F (*b*) and proband iPS-CMs (*c*). (*d–h*) The resting potential (*d*), AP amplitude (*e*), APD_90_ (*f*), APD_70_ (*g*) and APD_50_ (*h*) of the iPS-CMs. Data shown as mean fold change ± s.e.m., *n* = 6, *p < 0.05, **p < 0.01, ***p < 0.001, one-way ANOVA. (*i–l*) Typical early afterdepolarization (EAD) events illustrating the effect of changing activation frequency to 4 Hz in iPS-CMs from the family pedigree.

Confocal microscopy imaging was used to examine for spontaneous Ca^2+^ release events interspersed between Ca^2+^ transients normalized to baseline fluorescence, *F*/*F*_0_, in spontaneously beating iPS-CMs (figure 3). Under baseline conditions proband-M iPS-CMs generated Ca^2+^ signals with regular frequency and amplitude at 40–45 s intervals. All the proband-M iPS-CMs also showed normal Ca^2+^ transient signals with no occurrence of either PCTPTCs or COs (figure 2*a*); proband-F iPS-CMs carrying the RYR2-A1855D variant showed Ca^2+^ signals showing irregular diastolic deflections (figure 2*b*). Two such cells showed PCTPTC events, while eight cells showed normal Ca^2+^ handling. The proband iPS-CMs showed Ca^2+^ signals reflecting markedly disordered Ca^2+^ homeostasis. The traces showed multiple (greater than 3) EAD, events following the evoked Ca^2+^ transients whose amplitudes themselves were inconsistent (figure 2*c*). Two of the proband iPS-CMs showed COs; while the other cells showed normal evoked Ca^2+^ signals (figure 2*c*).

ISO (100 nM) challenge did not elicit PCTs or COs in the proband-M iPS-CMs (figure 2*h*). However, in both iPS-CMs with the RYR2-A1855D and iPS-CMs with the heterozygous RYR2-A1855D and SCN10A-1362H variants, ISO produced pronounced effects on the occurrence of diastolic Ca^2+^ transients. One of the proband-F iPS-CMs showed PCTPTCs and one showed COs. IPS-CMs of the proband showed increased higher frequencies of diastolic CO (4 out of 10 cells) after ISO application (figure 3*g*,*h*).

### (d) Electrophysiological features of the induced pluripotent stem cells-induced cardiomyocytes

APs were then recorded and analysed in iPS-CMs using whole cell patch electrodes on day 40 since their differentiation. The records of the AP waveforms (figure 3*a–c*) were used to obtain their resting potentials (RPs), AP amplitudes (APA) and AP duration at 90% full repolarization (APD_90_), APD_70_ and APD_50_. Firstly, RPs (figure 3*d*) and APAs (figure 3*e*) were statistically indistinguishable between the proband, F and M, iPS-CMs both before and following ISO treatment. Thus, RPs before compared with following ISO treatment in iPS-CMs of the proband-M were −68.92 ± 5.05 mV versus −68.62 ± 5.62 mV; of proband-F they were −63.42 ± 12.03 mV versus −67.70 ± 8.01 mV; of the proband they were −73.47 ± 3.21 mV versus −63.01 ± 15.78 mV (*p* > 0.05, *n* = 6 respectively). The APAs before and following ISO treatment, in the proband-M were 89.87 ± 12.40 mV and 93.86 ± 8.32 mV; in the proband-F they were 110.10 ± 10.89 mV and 92.29 ± 10.11 mV; and in the proband they were 95.14 ± 16.85 mV and 102.70 ± 15.32 mV (*p* > 0.05, *n* = 6, respectively).

Secondly, in terms of the AP waveform itself, the proband iPS-CMs showed the longest APDs compared with those of the proband-M and proband-F. Thus, before ISO challenge, the APD_90_ and APD_70_ of the proband iPS-CMs was markedly extended compared to that of the proband-M (215.60 ± 27.46 ms versus 135.0 ± 7.96 ms and 116.8 ± 14.64 ms versus 70.73 ± 9.32 ms, respectively, *p* < 0.05, *n* = 6). The APD_50_ of the proband iPS-CMs was increased relative to those of the proband-M (78.14 ± 6.504 ms versus 47.46 ± 9.46 ms, *p* > 0.05, *n* = 6). The APD_90_, APD_70_, APD_50_ of the proband-F iPS-CMs were not detectably altered compared to those of the proband-M (161.8 ± 17.04 ms versus 135.0 ± 7.96 ms, 84.97 ± 10.65 ms versus 70.73 ± 9.32 and 58.51 ± 12.74 ms versus 47.46 ± 9.45, *p* > 0.05, *n* = 6).

Thirdly, the AP waveforms appeared remodelled during ISO application with APD shortening in both the proband and the parents’ iPS-CMs. ISO application shortened APD_90_, APD_70_ and APD_50_ in the iPS-CMs of the proband and his parents (table 1). ISO application reduced the iPS-CM-M APD_90_ (*p* > 0.05, *n* = 6), APD_70_ (*p* > 0.05, *n* = 6), APD_50_ (*p* > 0.05, *n* = 6) but not to statistical significance. ISO decreased APD in the proband and proband-F iPS-CMs as shown in table 1. The APD_90_ (*p* < 0.01, *n* = 6), APD_70_ (*p* < 0.05, *n* = 6) and APD_50_ (*p* < 0.05, *n* = 6) of the proband-F iPS-CMs were significantly decreased. The APD_90_ (*p* < 0.001, *n* = 6), APD_70_ (*p* < 0.05, *n* = 6), APD_50_ (*p* < 0.05, *n* = 6) rangeability of the proband iPS-CMs were similarly decreased (table 1 and figure 3).

**Table 1.** Influence of ISO stimulation on AP characteristics.

	group	n	RP (mV)	APA (mV)	APD_90_ (ms)	APD_70_ (ms)	APD_50_ (ms)
baseline	proband-M	6	−68.92 ± 5.05	89.87 ± 12.40	135.0 ± 7.96	70.73 ± 9.32	47.46 ± 9.45
	proband-F	6	−63.42 ± 12.03	110.10 ± 10.89	161.81 ± 7.04	84.97 ± 10.65	58.51 ± 12.74
	proband	6	−73.47 ± 3.21	95.14 ± 16.85	215.60 ± 27.46^a^*	116.80 ± 14.64^a^*	78.14 ± 9.12
ISO (100 nM)	proband-M	6	−68.62 ± 5.62	93.86 ± 8.32	87.71 ± 9.36	47.60 ± 6.40	31.42 ± 5.42
	proband-F	6	−67.70 ± 8.01	92.29 ± 10.11	77.14 ± 8.57^b^**	36.80 ± 3.47^b^*	22.60 ± 3.19^b^*
	proband	6	−63.01 ± 15.78	102.70 ± 15.32	112.40 ± 15.69^c^***	47.98 ± 6.70^c^***	28.42 ± 6.50^c^**

^a^*p < 0.05, proband (baseline) compared with proband-M (baseline).

^b^*p < 0.05, **p < 0.01, proband-F (baseline) compared with proband-F + ISO.

^c^**p < 0.01, ***p < 0.001, proband (baseline) compared with proband + ISO.

Finally, we investigated the effect of altering the maintained stable pacing rate [32] from pacing at 1 Hz (60 bpm, close to normal resting human heart rate) [33]. Extreme increases in the pacing rate to 4 Hz were used to emulate iPS-CM responses to exercise and stress. EADs were not observed in the proband-M iPS-CMs (figure 3*j*). However, iPS-CMs of the proband-F revealed one episode of EAD in one cell and EAD occurred in two iPS-CMs of the proband. The results suggested that RYR2-A1855D and SCN10A-1362H double variants may increase the susceptibility of cardiomyocytes to arrhythmias.

## 4. Discussion

This study illustrates use of iPSCs to determine cardiomyocyte phenotypes in determination and analysis of clinical cardiac arrhythmogenic mechanisms in family pedigrees. It employed as an example, novel heterozygous RYR2-A1855D and SCN10A-Q1362H variants occurring alone and in combination. We had identified an infant with PVT episodes carrying heterozygous RYR2-A1855D and SCN10A-1362H variants. We successfully differentiated iPS-CMs of the family pedigree *in vitro*, and confirmed that the proband iPS-CMs with the double variant showed a pro-arrhythmic phenotype more severe than that of the parents. The experiments demonstrated the iPSC systems as showing physiological properties reported in other systems, and then implicated altered Ca^2+^ homeostasis in this effect. This could potentially involve both resting free cytosolic Ca^2+^ levels, [Ca^2+^]_i_ and systolic, and abnormal diastolic [Ca^2+^]_i_ increases accompanying AP activity reflecting the consequent influx of extracellular Ca^2+^ or Ca^2+^ release from intracellular stores [34].

Firstly, background or overall Ca^2+^ release [4,35] has previously been implicated in regulation of transcriptional activation and gene expression in reports modelling [Ca^2+^]_i_-oscillations [24,36]. Here, we report that the RYR2-A1855D variant downregulated *RYR2* mRNA expression in proband and proband-F iPS-CMs. It also resulted in pathological protein deposition, aggregation and formation [35].

Secondly, established reports associate aberrant SR Ca^2+^ release, including that occurring under conditions of pathological RYR2 function, with pro-arrhythmic DADs and EADs. At the whole heart level, the underlying increased Na^+^/Ca^2+^ exchange or L-type Ca^2+^ channel activation has thus been implicated in diastolic premature ventricular contractions [37]. The present studies demonstrated that RYR2-A1855D predisposed to such [Ca^2+^]_i_ abnormalities, and that this was exacerbated by the presence of the SCN10A-Q1362H variant. The latter was reflected in the increased severity of the resulting phenotype in the proband compared to the proband-F iPS-CMs. By contrast, the SCN10A-Q1362H variant alone in the proband-M iPS-CMs was without effect.

Thirdly, clinical evidence associates abnormalities in several independent *SCN10A* loci with altered cardiac conduction reflected in PR and/or QRS intervals, AF [13,14] and BrS, while not necessarily invoking monogenic roles of *SCN10A* variants in the latter conduction disease [15,37]. However, in contrast to the primarily Nav1.5 cardiomyocyte expression, Nav1.8 is often regarded as a neuronal as opposed to the cardiomyocyte Na^+^ channel, nevertheless potentially important in cardiac nerves and ganglionated plexi [16]. However, the implied distinction between cardiac and neuronal Nav isoforms and its potential pro-arrhythmic impacts are subjects of current debate [21,22]. In one report, both actions of the Nav1.8-blocker A-803467 and Nav1.8-based late Na^+^ current were undetectable in most electrophysiological characterizations of normal rabbit and human, atrial and ventricular, cardiomyocytes and human iPS-CMs. Furthermore, human atrial tissue, rabbit ventricular tissue and human iPS-CMs then showed low to absent cardiac *SCN10A* mRNA expression [38]. These findings appeared to exclude important direct Nav1.8 function in cardiomyocytes from healthy hearts. However, it could still permit extra-cardiac effects or enhancer–promotor interactions between *SCN10A* and *SCN5A* loci [39]. Furthermore, other studies did detect *SCN10A* mRNA in murine atrial and ventricular mouse myocardium although at much lower expression levels than SCN5A [40]. Na_v_1.8 then influenced late rather than peak Na^+^ currents, actions also detected in intracardiac neurons [17]. *SCN10A* variants may thereby cause intracellular Na^+^ overload and APD prolongation [41]. The resulting Na^+^ accumulation may lead to intracellular Ca^2+^ overload by increasing NCX mediated Ca^2+^ influx potentially causing pro-arrhythmic effects [42]. Selective, A-803467 block then prolonged PR interval and QRS complex duration, shortened APD and suppressed EADs [43]. The present study demonstrated more significant APD prolongations in proband iPS-CMs carrying both RYR2-A1855D and SCN10A-Q1362H than in proband-F iPS-CMs carrying only RYR2-A1855D, with unchanged APD in proband-M iPS-CMs carrying only SCN10A-Q1362H. In addition, upregulation of Nav1.8 mRNA and protein expression with pharmacologically demonstrable functional effects in both cardiac tissue and cardiomyocytes, accompanied both cardiac failure [44] and hypertrophy [19], suggesting direct cardiomyocyte roles in diseased and/or remodelled hearts.

Fourthly, cardiac AP waveforms vary with, thereby adapting to, altered heart rates following increased adrenergic drive during *in vivo* stress or exercise [8,45]. At the cellular level, they are correspondingly accompanied by altered APD and Ca^2+^ transient time courses [46]. Increased heart rate increases diastolic [Ca^2+^]_i_ through the resulting increased net [Ca^2+^] influx, and decreases efflux owing to the shorter diastolic intervals [47]. This may underly the rate-dependent APD adaptations [48]. In the present experiments, high frequency, 4 Hz, stimulation enhanced the occurrence of EADs in the proband and proband-F, but not the proband-M iPS-CMs.

## 5. Study limitations and future work

The present study using iPS-CMs has several limitations that are worth mentioning. By prolonging the duration of the AP, *I*_Na-L_ increases the probability of EADs, which may trigger arrhythmias. In addition, enhanced *I*_Na-L_ may result in NCX-mediated Ca^2+^ release events. These could promote depolarizing transient inward currents (*I*_ti_), resulting in similarly pro-arrhythmic DADs [49]. In addition, selective pharmacological inhibition of Nav1.8 is known to reduce late Na^+^ current, pro-arrhythmic diastolic Ca^2+^ release, as well as spontaneous APs. This led to suggestions that Nav1.8-dependent selective *I*_Na-L_ reduction could offer a novel antiarrhythmic therapeutic approach [50]. However, the maturity of iPS-CMs differentiated *in vitro* remains an important issue when using these as a platform for such studies. Future studies could use the Nav1.8-blocker A-803467 and selective late sodium current inhibitors (e.g. ranolazine, GS967 or GS6615) in different experiment platforms for PVT.

## 6. Conclusion

In summary, the study investigates acute abnormalities of myocardial Ca^2+^ handling caused by the double novel heterozygous RYR2-A1855D and SCN10A-Q1362H variants associated with a clinical pro-arrhythmic phenotype. It associated the RYR2-A1855D but not the SCN10A-Q1362H variant when either was expressed alone, with abnormal Ca^2+^ homeostasis and APD prolongation. However, the compound heterozygous RYR2-A1855D and SCN10A-Q1362H variants showed an accentuated Ca^2+^ homeostasis and APD phenotype, suggesting a possible basis for the clinical features of the originating patient family.

## Data Availability

Supporting data were uploaded as the electronic supplementary material [51]. The remaining raw data are available upon reasonable request to the corresponding author.
